# Methionine synthase polymorphisms (*MTR 2756* A>G and *MTR 2758 C>G*) frequencies and distribution in the Jordanian population and their correlation with neural tube defects in the population of the northern part of Jordan

**DOI:** 10.4103/0971-6866.73405

**Published:** 2010

**Authors:** Helmi Yousif Al Farra

**Affiliations:** Yarmouk University, Irbid, Jordan; Dalhousie University, Halifax, Nova Scotia, Canada

**Keywords:** Jordan, *MTR*, neural tube defects, polymorphism

## Abstract

**BACKGROUND:**

The human methionine synthase gene (*MTR*) is located on chromosome 1q43; it is of 105.24 kb and is made up of 33 exons. Methionine synthase is a cytoplasmic enzyme that requires methylcobalamin for activity and catalyzes the remethylation of homocysteine to methionine. In this reaction, the methyl group of 5-methyltetrahydrofolate is transferred to the enzyme bond cob(I) alamin to generate methylcobalamin, followed by the transfer of the methyl group to homocysteine to reform methionine.

**MATERIALS AND METHODS:**

The frequencies of the polymorphisms of *MTR* 2756A>G and *MTR* 2758C>G have been determined in this study in a sample of 491 individuals collected from all regions of Jordan and representing the Jordanian population. The different alleles and genotypes at the two polymorphic sites were identified using the Polymerase Chain Reaction - Restriction Fragment Length Polymorphism (PCR-RFLP) analysis.

**RESULTS:**

Showed that the percentages of the polymorphic alleles at the *MTR* 2756 position in the north, middle and south regions were 90.38, 92.65 and 83.69%, respectively, for the *MTR* 2756A allele, and were 9.61, 7.34 and 16.30%, respectively, for the *MTR* 2756G allele, with overall percentages in the whole Jordanian population of 90.73 and 9.27% for the *MTR* 2756A and *MTR* 2756G alleles, respectively. The percentages of the genotype *MTR* 2756AA were 82.90% in the northern region, 86.72% in the middle region and 71.73% in the southern region, and an overall percentage of *MTR* 2756AA in the whole Jordanian population was 83.50%. The frequencies of *MTR* 2756AG genotype in the northern, middle and southern regions were 14.95, 11.84 and 23.91%, respectively, with an overall percentage of 14.46% in the whole Jordanian population. The percentages of the genotype *MTR* 2756GG in the northern, middle and southern regions were 2.13, 1.42 and 4.34%, respectively, with an overall percentage of 2.04% in the whole Jordanian population. Only the wild type allele (C) of the *MTR* 2758C>G polymorphism was detected in this study. In addition, the association of *MTR* 2756A>G and *MTR* 2758C>G polymorphisms with the development of neural tube defects (NTDs) was examined using 17 cases of mothers from the northern part of Jordan, who gave birth to NTD affected children during the period of this study. Results showed no association between these two examined polymorphisms and the increase in maternal risk for giving birth to NTD children.

**CONCLUSION:**

results of this study recommend that examination should be done on larger populations to arrive at better conclusions. Also, more studies on gene–gene interaction should be done to examine the associations with NTDs.

## Introduction

The human methionine synthase gene (*MTR*) is located on chromosome 1q43; it is of 105.24 kb and is made up of 33 exons.[1,2] Also, it produces about 1265 amino acid residues and weighs 140.5 kDa.[3] Methionine synthase is a cytoplasmic enzyme that requires methylcobalamin for activity and catalyzes the remethylation of homocysteine to methionine. In this reaction, the methyl group of 5-methyltetrahydrofolate is transferred to the enzyme bond cob(I) alamin to generate methylcobalamin, followed by the transfer of the methyl group to homocysteine to reform methionine. In *MTR*, a polymorphism exists that is located at nucleotide position 2756 (*MTR 2756A>G*) and it changes an aspartic acid into a glycine.[4] *MTR 2758 C>G* is another polymorphism and it appears to have rare effects in the populations.[5] *MTR 2756A>G* polymorphism affects formyltetrahydropteroylglutamic acid (H4PteGlu) disposition of erythrocytes and the *MTR 2756AG* genotype is associated with more formyl-H4PteGlu, relative to 5-methyl-H4PteGlu, found in individuals with wild-type alleles. This relationship was not present in red blood cells of individuals with a neural tube defect (NTD).[6] The influence of *MTR 2756A>G* on total homocysteine plasma levels is still a matter of debate. An association was observed between *MTR 2756A>G* and increased total homocysteine levels,[7] while such an association was not confirmed in other studies.[6,8] Furthermore, an association between *MTR 2756A>G* and low plasma levels of homocysteine was observed.[9] Humans lacking *MTR* activity have severe clinical consequences.[10] In mice, complete loss of *MTR* activity leads to early embryonic lethality.[11] The polymorphism *MTR 2756A>G* as well as the heterozygous genotype *MTR 2756AG* was reported to be associated with the severity of coronary artery disease[12] and was considered as high risk factor for their occurrence.[13] Different studies, however, showed no association between *MTR 2756A>G* polymorphism and birth defects,[6,8] cerebrovascular, cardiovascular diseases,[14] and early onset vascular thrombosis.[15] The homozygous *MTR 2756GG* was however linked to a higher susceptibility for malignant lymphoma.[16]

## Materials and Methods

Blood samples were collected from 491 adult individuals representing the Jordanian population. These individuals were between 20 and 46 years of age and had no reported abnormalities; the samples were collected from different regions in Jordan according to the calculated sample sizes needed to represent the Jordanian population in the different regions. Most of the samples were taken from premarital testing centers, while some samples were collected from Yarmouk University campus and from parents accompanying children to the hospitals. Seventeen samples were collected from case mothers who had given birth to at least one NTD affected child from the largest two hospitals among eight hospitals having pediatric departments (King Abdullah and Princes Rahma hospitals), from the northern part of Jordan, between the beginning of September 2007 and the end of November 2008. All the volunteers signed a written consent.

Three milliliters of blood was collected from each subject in an ethylenediamine tetraacetic acid (EDTA) tube, and then genomic DNA was isolated from lymphocytes in the whole blood collected in EDTA vacutainers by using standard procedures.[17] A 176 base pair (bp) fragment (NCBI) of the *MTR* gene which includes the 2756 region was amplified using the forward (*MTR*) 5’-CATGGAAGAATATGAAGATATTAGAC-3’and the reverse (*MTR*) 5’-GAACTAGAAGACAGAAATTCTCTA–3’ (Midland, TX, USA) as described in Zhu *et al*.[5] the polymerase chain reaction (PCR) amplification was carried out for 38 cycles in a Gene-Amp 9600 PCR system (Perkin-Elmer) under the following conditions: denaturation at 95°C for 45 sec, annealing at 72°C for 35 sec, and extension at 72°C for 75 sec.[5]

Restriction enzyme digestion was performed using the *Hae*III restriction enzyme. Each restriction digestion reaction contained 10 μl of the PCR product, 10 units of *Hae*III (Biolabs, England), 10× NE buffer2 (Biolabs, England), 0.1 mg/ml bovine serum albumin (BSA) (Promega, USA), and sterile d.d. H_2_O made to a final volume of 20 μl. The reaction tube was then incubated at 37°C for 3 hours. The enzyme was then inactivated at 65°C for 15 minutes.[5]

The separation of the digested normal 2756A/A genotype on 3% agarose gels resulted in a single undigested fragment of 176 bp, while the mutant polymorphic homozygote genotype 2756G/G resulted in two fragments of 146 and 30 bp and the digestion of the heterozygous 2756A/G genotype produced three fragments of 176, 146 and 30 bp.[18]

Restriction enzyme digestion was performed using the *Sau*96I restriction enzyme on the same fragment as in the previous section. Each restriction digestion reaction mixture contained 10 μl of the PCR product, 10 units of *Sau*96I (Biolabs, England), 10× NEbuffer4 (Biolabs, England), 0.1 mg/ml BSA (Promega, USA), and sterile d.d. H_2_ O made to a final volume of 20 μl. The reaction tube was then incubated at 37°C for 3 hours. The enzyme was then inactivated at 65°C for 15 minutes.[5] The digestion of the normal 2758C/C genotype resulted in two fragments of 148 and 28 bp, while the mutant polymorphic 2758G/G resulted in one fragment of 176 bp. The digestion of the heterozygous 2758C/G genotypes produced three fragments of 176, 148 and 28 bp.[18]

Enumeration data including the number of individuals with various genotypes and the comparisons between regions were evaluated using the Chi-Square and Fisher’s-exact tests. *P* value equal to 0.05[19] was statistically significant and all the analysis had been done using the SPSS software version 15.5.

## Results

The frequencies of the different alleles and genotypes of the *MTR 2756* and *MTR 2758* positions were examined in 491 samples of the three regions of Jordan. However, the calculated minimal sample size that is representative to the whole of Jordan was 385.

**Figure 1:**
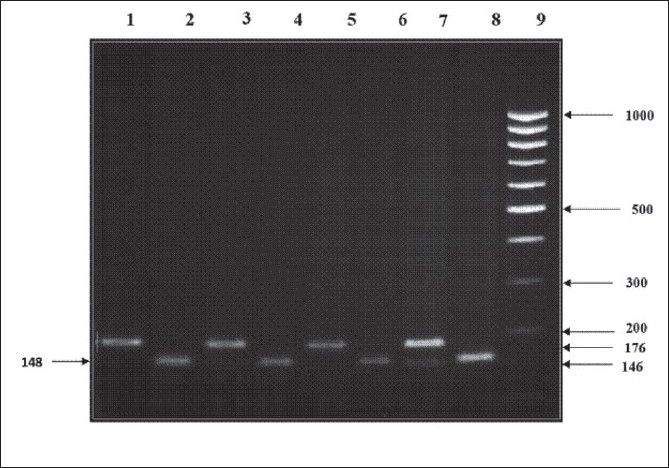
Restriction digestion products of representative polymorphisms of the MTR 2756 after digestion with HaeIII and *MTR 2758* after digestion with Sau96I and separation on 3% agarose gel. Lane 9: 100 bp DNA marker; lanes 2, 4, 6 and 8: homozygous *MTR 2758* CC normal genotype with 148 bp; lane 7: *MTR 2756* AG heterozygous genotype with 176 and 146 bp fragments; lanes 1, 3 and 5: normal homozygous *MTR 2756* AA genotype with one band of 176 bp.

Figure 1 shows the digestion products of the different genotypes found in our subjects. The amplified sample with of the normal *MTR 2756AA* genotype gave no sites for *Hae*III resulting in uncut amplified fragment with a size of 176 bp; the *MTR* fragment with the *MTR 2756GG* genotype gave two 146 and 30 bp long fragments; and the fragment with the *MTR 2756AG* genotype gave three 176, 146 and 30 bp long fragments. The amplified fragment of the normal *MTR 2758CC* genotype had one restriction site for the *Sau*96I enzyme, giving two fragments of length 148 and 28 bp 43, while the *MTR* fragment containing the polymorphic GG genotype remained intact with its original 176 bp length. The sample that had the CG genotype showed three fragments: the uncut wild type176 bp, and the products of the cut mutant allele of 148 and 28 bp long.

Allele percentage and frequencies of *MTR 2756A>G* in the different regions of Jordan are summarized in Table 1. The cross tabulation displays that the percentage of the A allele in the northern region was 90.38%, in the middle region was 92.65% and in the southern region (Aqaba) was 83.69%, while the percentage of the G allele was 9.61, 7.34 and 16.30% in the northern, middle and southern regions, respectively. A two-way contingency table analysis showed that there was a statistical correlation between the *MTR 2756A>G* alleles and the regions in Jordan (*P* = 0.025). The possible explanation is that the south region had the lesser allele percentage than the other regions.

**Table 1:** Percentages (frequencies) of *MTR 2756A>G* alleles in the three regions of Jordan

Regions	Allele A	Allele G
Northern region (468)	90.38% (423)	9.61% (45)
Middle region (422)	92.65% (391)	7.34% (31)
Southern region (92)	83.69% (77)	16.30% (15)

The percentages of the normal *MTR* (*A/G*) genotype were 82.90% in the northern region, 86.72% in the middle region and 71.73% in the southern region.

The percentages of the heterozygous *MTR 2756* (A/G) genotype were 14.95, 11.84 and 23.91% in the northern, middle, and southern regions, respectively. The percentages of the *MTR 2756* homozygous mutant G/G genotype were 2.13% in the northern region, 1.42% in the middle region and 4.34% in the southern region. A two-way contingency table analysis showed that there was no statistical association between the *MTR 2756A>G* genotypes and regions in Jordan (*P* = 0.163). The frequency of the *MTR 2758*C in all 491 studied samples was 100%, where no *MTR 2758*G allele was present in this study.

In an attempt to study the correlation between the two studied polymorphisms *MTR 2756A>G* and *MTR 2758C>G* and maternal risk for giving birth to children with NTD, a group of 17 mothers who gave birth to NTD affected children in the northern part of Jordan during the period of this study, from September 2007 to November 2008, were tested for the frequencies of these alleles and were compared with the control samples collected from the same region at the same time. The percentage and frequencies of *MTR 2756A* and *MTR 2756G* alleles in the NTD cases are shown in Table 2. The percentages of the A and G alleles in the case mothers were 82.35 and 17.64%, respectively, while in the controls they were 90.38 and 9.61%, respectively. The results of the Chi-square test for comparison between the allele frequencies in the control samples and case mothers indicated no association (*P* = 0.134) between these alleles and the incidence of NTD cases. Due to the fact that the expected value of Chi-square was more than 20%, Fisher’s exact test was used in comparing the percentages of genotypes in the control samples with those of the case mothers. This analysis showed that there were no correlations (P = 0. 107) between NTD cases and the *MTR 2756A>G* polymorphism.

**Table 2:** Genotypes and allele of *MTR 2756A>G* percentages and frequencies in the NTD affected mothers and control group from the north part of Jordan

Genotype	NTD	Controls	Allele	NTD	Control
AA	64.7% (11)	82.91% (194)	A	82.35% (28)	90.38% (423)
AG	35.3%	35% (35)	G	17.64% (6)	9.61% (45)
GG	0% (0)	2.13% (5)			
Total	100% (17)	100% (234)	Total	100% (34)	100% (468)

The *MTR 2758C>G* polymorphism was not found either in the NTD case mothers or in the controls.

## Discussion

This is the first study that has examined the frequencies and distribution of the polymorphisms of methionine synthase, *MTR 2756A>G* and *MTR 2758C>G*, in the Jordanian population and the Arab countries, except for one study which reported on a limited sample of 76 subjects in the city of Casablanca, Morocco, and examined the frequencies of alleles and genotypes of the polymorphism of *MTR 2756A>G* in normal individuals.[20]

Besides, this study has also tried to evaluate the correlation between the polymorphisms *MTR 2756A>G* and *MTR 2758C>G* and maternal risk of delivering NTD affected babies in the northern part of Jordan. These polymorphisms were examined due to their role in the folate metabolic pathway, which is essential for the methylation and regulation of developmental processes in embryos.[21] The etiology of NTD is poorly understood, but it is now suggested that there is a complex interplay between environmental and genetic factors.[22] Knowledge of the association between NTD and various genetic markers related to development may help increase our understanding of the genetics of NTD pathogenesis.

Based on the links between the *MTR* polymorphisms with folate metabolism pathway and the results of previous studies, we examined the frequencies of the two different polymorphisms, *MTR 2756A>G* and *MTR 2758C>G*, in the whole of the Jordanian population, and their relation to NTDs in the northern region of the country. The results of this study showed that the distribution of all genotypes of the *MTR 2756A>G* in the examined sample was in Hardy-Weinberg equilibrium (*P* ≤ 0.05). There was no association between the frequencies of *MTR 2756G>A* and *MTR 2758C>G* alleles and genotypes among the different regions of Jordan, which indicated no genetic differences between the residents of the different major regions of Jordan.

The results also showed that the overall percentages of *MTR 2756* genotype in Jordan were 83.5% for AA, 14.46% for AG and 2.04% for GG. This was relatively different from the genotype percentages reported in the small sample of the Moroccan population, which were 73.4, 21.9 and 4.7% for AA, AG and GG, respectively,[20] but more close to those genotype percentages reported in Alabama state of the United States of America, which were 83.4% AA, 15.4% AG and 1.2% GG,[23] and those of the Asians, including the Chinese, Korean and Japanese populations, where the average percentages of the different genotypes in these populations were 71.9% for AA, 23.3% for AG and 4.8% for GG.[14,16]

The results of this study showed that there was only the wild type allele *MTR 2758C*, and *MTR 2758G* mutation was absent in the examined sample of the Jordanian population, which indicated that this mutant allele, if present in the Jordanian population, would have a percentage of less than 0.2%. This is in agreement with the rare incidence of this mutation *MTR 2758C>G*, which was reported only in one Caucasian male who had failure to severe eczema, megaloblastic anemia, and methylmalonic aciduria[4] and is similar to the results reported in Texas, USA (Zhu *et al* 2003).

The fact that the results of this study showed no association between *MTR 2756A>G* and *MTR 2758C>G* polymorphisms and maternal risk for NTDs, in the northern part of Jordan, is in agreement with the absence of association between *MTR 2756A>G* polymorphism and NTDs in Alabama, USA,[18] the Irish[24] and the Netherland populations.[25]

Concerning our attempt to investigate the association between *MTR 2756A>G* and *MTR 2758C>G* polymorphisms with the maternal risk for NTDs, in the northern part of Jordan, during 1 year period study (as part of the requirements for the MSc degree), we ended up with a limited small sample of case mothers due to the incidence of 3.8 affected with NTDs in 1000 live births in Jordan and 1.5 per 1000 live births in the northern region in Jordan,[26–28] which may have possibly affected our conclusion. Further tests of more mothers of NTD affected children in the future would be essential to confirm our results. Finally, the results of this study recommend that examination should be done on larger populations to arrive at better conclusions. Also, more studies on gene–gene interaction should be done to examine the associations with NTDs.
